# Assessing the Predictive Impact of Preoperative Lactate Dehydrogenase to Albumin Ratio on Outcomes Following Coronary Artery Bypass Graft Surgery

**DOI:** 10.3390/jcm14020554

**Published:** 2025-01-16

**Authors:** Ozgur Baris, Canbolat Mert Holat, Mustafa Eren Tosun, Ulviye Serenay Yaman, Aysegul Durmaz, Mustafa Canikoglu, Oguz Omay, Sadan Yavuz

**Affiliations:** 1 Cardiovascular Surgery, School of Medicine, Kocaeli University, Kocaeli 41001, Turkey; mertholat1@gmail.com (C.M.H.); mustafaerentosunmd@gmail.com (M.E.T.); drayseguldurmaz@gmail.com (A.D.); mustafacanikoglu@gmail.com (M.C.); oguzomay@gmail.com (O.O.); sadanyavuz67@yahoo.com.tr (S.Y.); 2 School of Medicine, Kocaeli University, Kocaeli 41001, Turkey; serenaymn01@gmail.com

**Keywords:** lactate dehydrogenase to albumin ratio (LAR), mortality, postoperative complications, length of stay

## Abstract

**Background:** The lactate dehydrogenase to albumin ratio (LAR) is a novel inflammatory marker and a potential predictor of mortality in various conditions. No research has yet examined LAR’s impact on mortality in cardiac surgery patients. This study evaluated LAR’s role in predicting mortality and complications in isolated coronary artery bypass grafting (CABG) patients. **Methods:** A retrospective analysis of 377 CABG patients (93 women, 24.7%; 284 men, 75.3%; mean age 65.9 years) from 2020 to 2024 was conducted. Data included demographics, preoperative characteristics, surgical details, and postoperative outcomes, along with ICU and hospital length of stay (LOS). **Results:** In-hospital mortality was 6.1% (*n* = 23). Independent predictors were low preoperative ejection fraction (EF) (OR: 0.96, *p* = 0.024), baseline LAR (OR: 1.08, *p* = 0.000), LOS-ICU (OR: 1.1, *p* = 0.000), postoperative ventricular tachycardia (OR: 37.9, *p* = 0.006), and acute renal failure (OR: 12.1, *p* = 0.000). Mortality cases had a higher median LAR than survivors (8.6 vs. 5.2, *p* = 0.000). Elevated LAR correlated with lower preoperative EF (r = −0.227, *p* = 0.000), longer LOS-ICU (r = 0.17, *p* = 0.001), and longer LOS-hospital (r = 0.208, *p* = 0.000). A LAR cut-off of 7.097 predicted mortality (AUC: 0.823, sensitivity 78.3%, specificity 77.1%). Elevated LAR values were observed in all groups with postoperative complications (*p* < 0.05), indicating its consistent association with negative outcomes. **Conclusions:** LAR is a valuable predictor of in-hospital mortality and postoperative complications in CABG patients. Elevated LAR is associated with longer ICU/hospital stays and poorer outcomes. Preoperative LAR assessment can guide risk stratification, forecast mortality, and inform surgical planning and treatment strategies.

## 1. Introduction

Coronary artery disease, a progressive condition often caused by familial predisposition and smoking, intertwined with systemic pathologies ranging from insulin resistance and hypertension to metabolic syndrome and diabetes, represents a complex clinical entity. Coronary artery bypass grafting (CABG) is an established therapeutic intervention for this disease. Despite advancements in technology, innovative surgical approaches and techniques, and improved cardiopulmonary bypass and perfusion methods, CABG still has mortality and morbidity that require significant focus. Consequently, preoperative patient management and the evaluation of risk factors continue to be areas of interest for clinical research.

Many CABG patients tend to be older, with a considerable number having previously experienced myocardial infarction (MI), stroke, or other health issues. These factors contribute to elevated rates of mortality and morbidity within this group of patients [1]. Recognizing factors that predict mortality and morbidity after CABG early on allows for the optimization of preoperative risks and the implementation of preventive strategies for high-risk individuals. In this regard, various biomarkers, including serum lactate dehydrogenase (LDH) and albumin levels, have become subjects of interest for research [2,3,4,5,6]. LDH, an enzyme crucial in glycolysis, facilitates pyruvate-to-lactate conversion and acts as an indicator of tissue and organ hypoperfusion [2]. Liu et al. demonstrated that elevated serum LDH levels independently predict 30-day mortality in sepsis patients [3]. In the context of cardiac surgery, preoperative LDH levels have been identified as independent predictors of in-hospital mortality and postoperative complications [4]. Albumin, a primary blood protein produced by the liver, plays significant roles in antioxidant activity, anti-inflammatory response, and plasma oncotic pressure regulation. A recent study has shown that reduced serum albumin levels are linked to increased mortality risk in critically ill patients [5]. Additionally, a meta-analysis revealed that hypoalbuminemia correlates with all-cause mortality and heightened risk of postoperative complications, such as extended stays in the intensive care unit (ICU) and hospital following cardiac surgery [6].

The lactate dehydrogenase (LDH) to albumin ratio (LAR) has been identified as a novel indicator of inflammation. By incorporating elements such as organ dysfunction, chronic conditions, inflammatory processes, and nutritional state, LAR offers more comprehensive prognostic insights compared to the individual predictive capabilities of LDH or albumin [2]. Studies on LAR have primarily examined its correlation with outcomes in cancer [7,8], infections of the lower respiratory tract [9], and severe infectious diseases [10]. Various factors, such as cancer, liver disorders, poor nutrition, and infections, can affect the levels of LDH and albumin. As a result, LAR is thought to be potentially more useful than either LDH or albumin alone in identifying patients who are critically ill. In COVID-19 patients, elevated LAR values have been correlated with extended hospitalization periods, increased ICU admissions, and higher death rates [11]. Furthermore, a study exploring the connection between LAR and prognosis in patients with sepsis-induced acute kidney injury found that high LAR values were linked to increased mortality rates at 28 days, 90 days, and during hospital stays [12]. Likewise, an elevated LAR value (≥10.57) has been associated with overall mortality in sepsis patients in the ICU, indicating its potential as a prognostic marker [2]. For patients with ARDS, a significant association has been demonstrated between high LAR and mortality rates at 30 days, 90 days, and during hospitalization [13].

Despite the increasing interest in utilizing LAR as a prognostic marker, to the best of our knowledge, no studies have evaluated the predictive effect of LAR on postoperative complications and mortality in patients undergoing cardiac surgery. This study aimed to assess the predictive effect of LAR on in-hospital mortality and postoperative complications in patients undergoing isolated CABG.

## 2. Materials and Methods

### 2.1. Study Population

A retrospective analysis was conducted on the medical records of 412 adult patients who underwent isolated coronary artery bypass graft (CABG) surgery at the Cardiovascular Surgery Department of Kocaeli University Faculty of Medicine Hospital from January 2020 to July 2024. This study ultimately included 377 patients after excluding those lacking initial laboratory measurements such as LDH and/or albumin. Additionally, patients who had urgent CABG surgeries and who had surgeries other than isolated CABG, including valve/multiple valve procedures and combined coronary + valve + aortic operations, were omitted from the study.

The Local Ethics Committee of Kocaeli University (approval number KÜ GOKAEK-2024/18.5, project number 2024/439, and approval date of 6 October 2024) approved this study, which adhered to the principles outlined in the Declaration of Helsinki. Given the retrospective nature of the research, obtaining informed consent was not necessary.

### 2.2. Data Collection

Patient medical records were analyzed under four sub-categories: demographic information, preoperative characteristics, surgical procedure specifics, and postoperative complications. Demographic data encompassed age, gender, smoking status, and comorbidities. Preoperative information included left ventricular ejection fraction (EF) percentage and the European System for Cardiac Operative Risk Evaluation (EuroSCORE) risk score. All phases, from admission to surgery and discharge, adhered to established guidelines. Surgical procedure details covered the surgical technique (off-pump coronary artery bypass (OPCAB) or on-pump cardiopulmonary bypass (CPB)), number of bypass grafts, CPB duration, cross-clamp time, and extubation period. Additionally, the duration of stays in the ICU and hospital were documented. Postoperative complications included atrial fibrillation (AF), ventricular tachycardia (VT), cerebrovascular disease (CVD), acute renal failure (ARF), pneumonia, and in-hospital mortality.

Standard preoperative laboratory tests, including complete blood count and biochemical analyses such as CRP, procalcitonin, LDH, albumin, and LAR, were also recorded. The LAR was calculated by dividing the LDH concentration by the albumin concentration. On the second day after admission, fasting blood samples were collected at 8:00–10:00 to measure hematological and biochemical indicators. A Sysmex XN-10 automatic hematology analyzer was used for complete blood count analysis (Sysmex CorporationTM, Kobe, Japan). Biochemical parameters were analyzed using a Cobas 8000 e 801 modular immunochemistry autoanalyzer (Roche Diagnostics GmbH, Mannheim, Germany).

### 2.3. Statistical Analysis

Statistical analyses were conducted using IBM SPSS Statistics for Windows version 25.0 (SPSS, Chicago, IL, USA). Numbers (percentages) were used to express categorical variables, while continuous variables were presented as medians (25th–75th percentiles) or mean ± standard deviation (SD). Categorical variables were compared using the chi-square test. The Kolmogorov–Smirnov test was used to evaluate the normality of numerical data. *t*-tests were applied for normally distributed data, while the Mann–Whitney U test was used for non-normally distributed data. To estimate crude and adjusted odds ratios (aORs) and 95% confidence intervals (CIs) for in-hospital mortality, univariate and binary logistic regression analyses (Enter method) were performed. The Spearman’s correlation coefficient was used to assess correlations between variables. The diagnostic performance of LAR was determined based on the receiver operating characteristic curve (ROC) and positive and negative predictive values. A post hoc power analysis was conducted utilizing G*Power 3.1.9.4 software. The significance level (α) was set at 0.05. Using our results obtained for LAR, the effect size was obtained as 0.77, and the power of our study was calculated to be 95%.

A two-sided *p*-value < 0.05 was considered statistically significant.

## 3. Results

### 3.1. Baseline Characteristics

The study population consisted of 377 patients who underwent isolated CABG, with 93 women (24.7%) and 284 men (75.3%). The mean age was 65.9 ± 8.6 years. Among the participants, 193 (51.2%) had a history of smoking, and 355 (94.2%) presented with comorbidities. The most prevalent comorbidities were hypertension (69%), diabetes mellitus (46.2%), and preoperative myocardial infarction (35.5%). According to the EuroSCORE, 95.5% of patients were classified as low risk. OPCAB was the surgical technique employed in 28.6% of cases, while CPB was used for the remainder. The median time to extubation was 9 h. For the 269 patients who underwent CPB surgery, the mean CPB and cross-clamp durations were 122.3 ± 35.7 and 70.6 ± 22 min, respectively. The median ICU and hospital stays were 4 and 8 days, respectively. Postoperative complications occurred in 133 patients (35.3%); AF (13.3%) and pneumonia (10.6%) were the most common postoperative complications. The baseline characteristics and the univariate analysis of in-hospital mortality are shown in Table 1.

### 3.2. Univariate Analysis of In-Hospital Mortality

The in-hospital mortality rate was 6.1% (*n* = 23) in isolated CABG patients. Analysis of baseline characteristics showed no significant differences in gender, age, or tobacco use between survivors and non-survivors (*p* > 0.05). While the presence of any comorbidity did not significantly correlate with in-hospital mortality, a subgroup analysis revealed that patients with preoperative MI (*p* = 0.000), CVD (*p* = 0.000), chronic renal disease (CRD) (*p* = 0.001), and cancer (*p* = 0.04) were significantly more prevalent in the mortality group compared to the survival group. Regarding preoperative factors, the mortality group exhibited significantly lower EF (%) (*p* = 0.000) and a higher proportion of high-risk patients, according to EuroSCORE (*p* = 0.01).

In terms of surgical procedures, a significant difference was observed in the surgical technique used (*p* = 0.036), but the number of grafted vessels did not differ significantly (*p* = 0.56). The in-hospital mortality group had significantly longer mean CPB time (163.5 ± 80.5 vs. 120.3 ± 31.2; *p* = 0.000) and cross-clamp time (85.8 ± 31.5 vs. 69.9 ± 21.3; *p* = 0.015). Additionally, the mortality group required longer extubation time (15 vs. 9; *p* = 0.000).

Among postoperative complications, VT (13% vs. 0.3%; *p* = 0.000) and ARF (39.1% vs. 4.8%; *p* = 0.000) were significantly higher in the mortality group. Additionally, this group had a longer median ICU stay duration (6 vs. 4; *p* = 0.026).

Analysis of baseline laboratory parameters using univariate methods revealed several markers associated with in-hospital mortality. Elevated levels were observed in leukocytes (*p* = 0.009), RDW (*p* = 0.004), creatinine (*p* = 0.002), LDH (*p* = 0.000), LAR (*p* = 0.000), procalcitonin (*p* = 0.02), and BNP (*p* = 0.000). In contrast, decreased levels of lymphocytes (*p* = 0.008) and albumin (*p* = 0.000) were also linked to higher in-hospital mortality rates (Table 1).

### 3.3. Binary Logistic Regression Analysis of In-Hospital Mortality

Binary logistic regression analysis was used to assess in-hospital mortality following isolated CABG. For modeling, variables that were significant in the univariate analysis were included, while only one of the potentially confounding variables (e.g., chronic renal disease and serum creatinine or albumin level; operation type and CPB time or cross-clamp time; preoperative MI history and serum BNP level) considered more critical was included to avoid overlap. The incidence of in-hospital mortality after isolated CABG was increased 37.9-fold (95% CI 2.89–496.7, *p* = 0.006) in the presence of postoperative VT; 12.1-fold (95% CI 3.027–48.527, *p* = 0.000) with postoperative ARF; 0.96-fold (95% CI 0.92–0.99, *p* = 0.024) with lower preoperative EF (%); 1.1-fold (95% CI 0.64–7.9, *p* = 0.000) with length of stay in ICU; and 1.08-fold (95% CI 1.05–1.15, *p* = 0.000) with higher LAR value (Table 2).

### 3.4. LDH/Albumin Ratio (LAR)

This study demonstrated that LAR was an independent predictor of in-hospital mortality. The median LAR value was significantly higher in the mortality group compared to the survivors [8.6 (7.1–13.2) vs. 5.2 (4.3–6.9); *p* = 0.000]. No statistically significant differences in median LAR values were observed with respect to demographic characteristics such as sex, comorbidities, and EuroSCORE risk. Moreover, the median LAR values were significantly elevated in all patients with postoperative complications. Table 3 presents a comparison of median LAR values according to demographic characteristics and the presence of postoperative complications.

Positive correlations were observed between LAR and age (r = 0.161; *p* = 0.002), LOS-ICU (r = 0.17; *p* = 0.001), and LOS-hospital (r = 0.208; *p* = 0.000). Additionally, a negative correlation was identified between LAR and preoperative EF (%) (r: −0.023; *p* = 0.000) (Table 4).

### 3.5. ROC Curve Analysis

**Figure 1:**
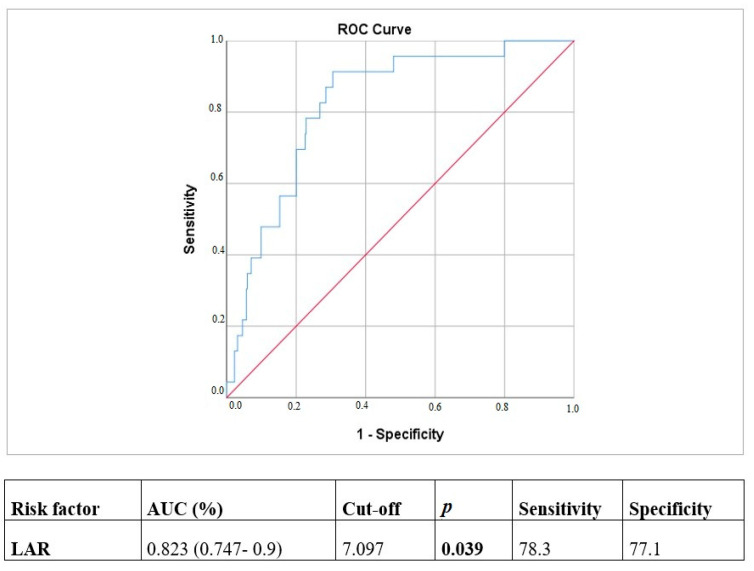
ROC analysis of LAR.

ROC analysis was conducted to assess the sensitivity and specificity of the LAR value in predicting in-hospital mortality. The optimal cut-off value for LAR in predicting mortality was determined to be 7.097 (area under the curve [AUC], 0.823, 95% confidence interval [CI]: 0.747–0.9; *p* = 0.039). The sensitivity and specificity were 78.3% and 77.1%, respectively (Figure 1).

## 4. Discussion

In this investigation, we determined that low preoperative EF (%), baseline LAR value, LOS in the ICU, postoperative VT, and postoperative ARF were independent predictors of in-hospital mortality in patients undergoing isolated CABG. Subjects in the mortality group demonstrated a significantly higher median LAR value compared to survivors. Furthermore, analysis of median LAR levels in relation to postoperative complications revealed markedly elevated LAR values across all groups that experienced complications. An inverse correlation was observed between LAR and preoperative EF (%), whereas positive associations were noted between LAR and length of stay in the ICU and hospital. For mortality prediction, the LAR cut-off value was determined to be 7.097 (AUC, 0.823; 95% CI: 0.747–0.9; *p* = 0.039), with sensitivity and specificity of 78.3% and 77.1%, respectively.

Despite recent advancements, mortality and morbidity after CABG remain significant clinical concerns. A meta-analysis has shown that advanced age, female sex, comorbidities such as diabetes mellitus (DM) and hypertension (HT), a history of preoperative MI, and previous cardiac surgery are associated with increased mortality after CABG surgery [1]. In our study, while no significant association was observed between sex or age and mortality, low preoperative EF (%), preoperative LAR value, prolonged stay in ICU, and the presence of postoperative VT and ARF were identified as factors associated with mortality following CABG.

For patients undergoing such a critical operation, predicting perioperative and postoperative complications and mortality and planning appropriate treatment strategies are crucial. To address this need, previous investigations have assessed specific blood parameters, including serum albumin and LDH levels [2,14,15,16]. Albumin, synthesized in the liver, is the main protein that constitutes the oncotic pressure of the blood. Also, it has anti-inflammatory, antioxidant, and antithrombotic properties [14]. Albumin, a negative acute phase reactant, plays an important role in systemic inflammation [2]. The typical serum albumin range for adults is 3.5 to 5.0 g/dL. Hypoalbuminemia is generally defined as a serum albumin level below 3.5 g/dL [15]. Studies have shown that hypoalbuminemia is a robust predictor of mortality in acute coronary syndrome patients, even after excluding confounding variables [15]. Research by Fındık et al. revealed that low preoperative albumin levels were linked to postoperative acute kidney injury and mortality in patients undergoing isolated CABG [16].

During cardiopulmonary bypass (CPB), the contact of blood with non-endothelial surfaces leads to a systemic inflammatory response, ischemia–reperfusion injury, and hemolysis. This inflammatory response triggers a strong thrombotic stimulus, and it also results in the production and release of various vasoactive and cytotoxic substances into the bloodstream. As a result of hemolysis, intracellular contents of red blood cells, such as hemoglobin and LDH, are released into the plasma [17]. LDH, a vital intracellular enzyme, plays a crucial role in energy production by facilitating the transformation of pyruvate to lactate under anaerobic conditions, which are essential for anaerobic metabolism. In a study by Zeng et al., preoperative LDH levels were found to be an independent variable in predicting postoperative complications in patients undergoing cardiac surgery. Furthermore, preoperative LDH levels were shown to be a stronger predictor of in-hospital mortality than other parameters, such as the neutrophil–lymphocyte ratio or lactate levels [4].

Serum albumin and LDH levels are routinely evaluated preoperatively and serve as appropriate monitoring parameters. Consequently, when these levels are found to be low in the preoperative period, it is essential to reassess them prior to discharge to address the underlying etiological cause [14]. Hypoalbuminemia may result from various underlying factors, including reduced protein intake (malnutrition, malabsorption), decreased hepatic synthesis, increased protein loss (hemorrhage, nephrotic syndrome, protein-losing enteropathy), elevated catabolic rates (infection, sepsis, cancer, etc.), and inflammation [2]. Therefore, rather than evaluating albumin or LDH levels independently, combining these two parameters may prove more efficacious.

The LDH-to-albumin ratio (LAR) serves as a novel inflammatory marker, offering more comprehensive prognostic information than LDH or albumin individually [2]. Researchers have explored LAR’s predictive value across various health conditions, including cancer [7,8], respiratory infections [9], severe infectious diseases [10,11], sepsis [12], and ARDS [13]. Hu et al. reported that LAR was a prognostic indicator for 30-day mortality in acute pulmonary embolism patients [18]. A positive association was found between LAR and mortality rates in both ICU and hospital settings for patients with septic myocardial injury [19]. Furthermore, Alizadeh et al. reported that elevated LAR levels correlated with extended hospital stays and increased ICU admissions among COVID-19 patients [11]. While numerous studies have examined the separate effects of LDH and albumin on mortality in isolated CABG patients, our extensive literature review reveals a lack of research investigating LAR’s prognostic significance in this specific context. In line with previous findings, our study demonstrated that LAR independently predicted in-hospital mortality for isolated CABG patients. We observed a positive correlation between LAR and the duration of ICU and hospital stays, consistent with Alizadeh et al.’s findings. To our knowledge, no prior research has assessed the relationship between LAR and postoperative complications. Our study found significantly higher median LAR levels in all postoperative complication groups compared to those without complications, representing a unique and distinctive aspect of this research.

The area under the curve (AUC) value for LAR was [0.63, (95% CI: 0.58–0.67)] in a study evaluating the impact of LAR on mortality in sepsis-related myocardial injury (19). In a retrospective cohort including patients with ARDS, the AUC value for LAR was [0.694 (95% CI: 0.634–0.754, *p* < 0.001)] [13]. In this study, the AUC value for LAR [0.823, 95% CI: 0.747–0.9, *p* = 0.039] was superior to that reported in the literature.

In the study conducted by Hu and Zu, ROC curve analysis demonstrated that the cut-off value of LAR for predicting 30-day mortality was 8.349, with a sensitivity of 80% and a specificity of 67% [18]. Consistent with this investigation, the cut-off value of LAR for predicting in-hospital mortality was determined to be 7.097 in our study. The sensitivity and specificity were 78.3% and 77.1%, respectively.

The present study had several limitations. Firstly, its retrospective design and the prognostic impact of the LAR were assessed solely for in-hospital mortality. Furthermore, the LAR value was evaluated exclusively during the preoperative period, and the efficacy of follow-up values in the postoperative period was not investigated. Multicenter prospective studies are necessary to evaluate the prognostic efficacy of LAR in isolated CABG patients, particularly its impact on long-term mortality.

Despite its constraints, this investigation possesses considerable merits. While previous research has independently demonstrated the prognostic value of LDH and albumin levels in cardiac surgery patients, our study is, to the best of our knowledge, the first to examine the influence of LAR on mortality in this specific cohort. The research distinguishes itself through its comprehensive approach, not only assessing mortality but also exploring the impact of LAR on postoperative complications, duration of ICU stay, and overall hospital length of stay, thereby contributing a robust and innovative perspective to the field.

A thorough evaluation of a patient’s risk factors before surgery serves multiple purposes. It not only aids in predicting potential surgical complications and mortality rates but also guides the selection of the most suitable surgical technique. These techniques may encompass minimally invasive operations, endovascular and hybrid methods, as well as on-pump or off-pump coronary procedures. For patients deemed high-risk, less invasive approaches might be preferable, whereas conventional surgical methods could be more appropriate for those categorized as low-risk.

## 5. Conclusions

In conclusion, this pioneering study evaluated the prognostic value of LAR for mortality and postoperative outcomes in patients who underwent isolated CABG. LAR was found to be a significant predictor of in-hospital mortality in this patient population. Furthermore, LAR has been shown to have a strong association with postoperative complications and prolonged intensive care and hospital stays. Preoperative LAR assessment may be considered an important predictor for identifying high-risk isolated CABG patients, predicting mortality and adverse outcomes, and subsequently determining surgical and treatment decisions.

## Figures and Tables

**Table 1:** Baseline characteristics and univariate analysis of in-hospital mortality of isolated CABG patients (*n* = 377).

		Total (*n* = 377)	In-Hospital Mortality (*n* = 23)	In-Hospital Survivors (*n* = 354)	*p*
*Demographic characteristics*					
Gender	Women Men	93 (24.7%) 284 (75.3%)	9 (39.1%) 14 (60.9%)	84 (23.7%) 270 (76.3%)	0.09
Age (year)	Mean ± SD	65.9 ± 8.6	68.7 ± 9.1	65.7 ± 8.6	0.11
Smoking history, *n* (%)	Non-smoker Current smoker Former smoker	184 (48.8%) 89 (23.6%) 104 (27.6%)	14 (60.9%) 4 (17.4%) 5 (21.7%)	170 (48%) 85 (24%) 99 (28%)	0.49
Comorbidity	(+) (−)	355 (94.2%) 22 (5.8%)	22 (95.7%) 1 (4.3%)	333 (94.1%) 21 (5.9%)	0.75
	Hypertension Myocardial infarction Congestive Heart Failure Peripheral Arterial Disease Cerebrovascular Disease COPD Diabetes Mellitus Chronic Renal Disease Malignity	260 (69%) 134 (35.5%) 30 (8%) 28 (7.4%) 44 (11.7%) 27 (7.2%) 174 (46.2%) 37 (9.8%) 36 (9.5%)	15 (65.2%) 16 (69.6%) 3 (13%) 1 (4.3%) 8 (34.8%) 2 (8.7%) 7 (30.4%) 7 (30.4%) 5 (21.7%)	245 (69.2%) 118 (33.3%) 27 (7.6%) 27 (7.6%) 38 (10.2%) 25 (7.1%) 167 (47.2%) 30 (8.5%) 31 (8.8%)	0.69 0.000 0.35 0.56 0.000 0.77 0.12 0.001 0.04
*Preoperative characteristics*					
EuroSCORE, *n* (%)	Low risk Moderate risk High risk	360 (95.5%) 13 (3.4%) 4 (1.1%)	20 (87%) 1 (4.3%) 2 (8.7%)	340 (96%) 12 (3.4%) 2 (0.6%)	0.01
Preoperative EF, (%)	Mean ± SD	55.9 ± 12.45	43.9 ± 13.2	56.7 ± 12.01	0.000
*Surgical procedure*					
Surgical technique, *n* (%)	OPCAB CPB	108 (28.6%) 269 (71.4%)	11 (47.8%) 12 (52.2%)	97 (27.4%) 257 (72.6%)	0.036
CPB time, hour	Mean ± SD	122.3 ± 35.7	163.5 ± 80.5	120.3 ± 31.2	0.000
Cross clamp time, hour	Mean ± SD	70.6 ± 22	85.8 ± 31.5	69.9 ± 21.3	0.015
Extubation time, hour	Median/(25th–75th percentiles)	9 (7–13)	15 (12–36)	9 (7–13)	0.000
Number of grafts	1 2 3 4 5 6 7	9 (2.4%) 42 (11.1%) 132 (35%) 135 (35.8%) 50 (13.3%) 8 (2.1%) 1 (0.3%)	2 (8.7%) 3 (13%) 7 (30.4%) 8 (34.8%) 3 (13%) 0 0	7 (2%) 39 (11%) 125 (35.3%) 127 (35.9%) 47 (13.3%) 8 (2.3%) 1 (0.3%)	0.56
*Postoperative complications*					
	Postoperative AF Postoperative VT Postoperative CVD Postoperative ARF Postoperative pneumonia	50 (13.3%) 4 (1.1%) 4 (1.1%) 26 (6.9%) 49 (10.6%)	6 (26.1%) 3 (13%) 1 (4.3%) 9 (39.1%) 3 (13%)	44 (12.4%) 1 (0.3%) 3 (0.8%) 17 (4.8%) 37 (10.5%)	0.06 0.000 0.11 0.000 0.7
LOS-ICU, day	Median (25th–75th percentiles)	4 (3–5)	6 (3–14)	4 (3–5)	0.026
LOS-hospital, day	Median (25th–75th percentiles)	8 (7–11)	7 (3–19)	8 (7–11)	0.41
*Preoperative routine laboratory tests*					
WBC, × 10^3^,	Median/(25th–75th percentiles)	7.9 (6.7–9.5)	7.7 (6.5–9.2)	7.9 (6.7–9.59)	0.6
Leukocyte, × 10^3^	Median/(25th–75th percentiles)	4.9 (3.9–6.4)	4.8 (4–7)	4.9 (3.9–6.3)	0.56
Leukocyte, %	Mean ± SD	63.5 ± 10.2	68.9 ± 11.5	63.1 ± 10.1	0.009
Lymphocyte, × 10^3^	Median/(25th–75th percentiles)	1.9 (1.3–2.4)	1.1 (0.9–2.2)	1.9 (1.4–2.4)	0.008
Lymphocyte, %	Mean ± SD	24.6 ± 8.9	20.1 ± 9.2	24.9 ± 8.8	0.01
Hemoglobin, (g/dL)	Mean ± SD	12.89 ± 2.04	12.1 ± 2.2	12.95 ± 2.01	0.055
Hematocrit	Mean ± SD	37.7 ± 5.6	36.2 ± 5.9	37.8 ± 5.6	0.2
Platelet, × 10^3^	Mean ± SD	242,654 ± 66,902	246,443 ± 79,519	242,408 ± 66,125	0.78
MPV, (fL)	Mean ± SD	9.96 ± 1.2	9.55 ± 1.3	9.99 ± 1.2	0.076
RDW-cv	Median/(25th–75th percentiles)	13.6 (12.9–14.7)	14.3 (13.7–16.2)	13.6 (12.8–14.6)	0.004
Creatinine	Median/(25th–75th percentiles)	0.9 (0.8–1.1)	1.1 (0.9–3.3)	0.9 (0.8–1.1)	0.002
AST	Median/(25th–75th percentiles)	20.7 (16–30.3)	20.3 (16.7–30.5)	20.8 (15.9–30.3)	0.76
ALT	Median/(25th–75th percentiles)	17 (12.3–25.5)	16.9 (13–22.5)	17 (12.3–26)	0.65
Total bilirubin	Median/(25th–75th percentiles)	0.44 (0.3–0.7)	0.6 (0.4–0.9)	0.4 (0.3–0.7)	0.15
Indirect bilirubin	Median/(25th–75th percentiles)	0.26 (0.2–0.4)	0.3 (0.2–0.6)	0.3 (0.2–0.4)	0.29
LDH, (U/L)	Median/(25th–75th percentiles)	211 (172–269)	367 (240–489)	207 (171–262)	0.000
Albumin, (g/L)	Mean ± SD	39.02 ± 5.5	35.04 ± 6.8	39.3 ± 5.3	0.000
LAR	Median/(25th–75th percentiles)	5.35 (4.3–7.14)	8.6 (7.1–13.2)	5.2 (4.3–7)	0.000
CRP, (mg/L)	Median/(25th–75th percentiles)	6.1 (2–22)	6.1 (2–16)	6.1 (2–22)	0.85
Procalcitonin, (ng/mL)	Median/(25th–75th percentiles)	0.12 (0.05–0.6)	0.76 (0.12–4.1)	0.12 (0.05–0.4)	0.02
BNP	Median/(25th–75th percentiles)	642 (173.5–2455)	7310 (4474–17,114)	537 (163–2103)	0.000

Abbreviations: SD: Standard deviation; COPD: Chronic obstructive pulmonary disease; EF: Ejection fraction; OPCAB: Off-pump coronary artery bypass; CABG: On-pump cardiopulmonary bypass; AF: Atrial fibrillation; VT: Ventricular tachycardia; CVD: Cerebrovascular disease; ARF: Acute renal failure; LOS: Length of stay; ICU: Intensive care unit; WBC: White blood cell; MPV: Mean platelet volume; RDW: Red cell distribution width; AST: Aspartate aminotransferase; ALT: Alanine aminotransferase; LDH: Lactate dehydrogenase; LAR: LDH/albumin ratio; CRP: C-reactive protein; BNP: Brain natriuretic peptide.

**Table 2:** In binary logistic regression analysis: risk factors associated with in-hospital mortality of isolated CABG patients.

Variable	OR [95% CI]	*p*
Preoperative MI	2.25 [0.64–7.9]	0.21
Cerebrovascular disease	1.8 [0.44–7.41]	0.4
Chronic renal disease	2.42 [0.59–9.9]	0.22
Malignity	2.54 [0.58–11.03]	0.22
Preoperative EF (%)	0.96 [0.92–0.99]	0.024
Operation type (OPCAB/CPB)	0.44 [0.13–1.5]	0.19
Postoperative VT	37.9 [2.89–496.7]	0.006
Postoperative ARF	12.1 [3.027–48.527]	0.000
LOS-ICU	1.1 [0.64–7.9]	0.000
LAR	1.08 [1.05–1.15]	0.034
RDW	1.1 [0.92–1.35]	0.29

Abbreviations: MI: Myocardial infarction; EF: Ejection fraction; OPCAB: Off-pump coronary artery bypass; CPB: On-pump cardiopulmonary bypass; VT: Ventricular tachycardia; ARF: Acute renal failure; LOS: Length of stay; ICU: Intensive care unit; LAR: LDH/albumin ratio; RDW: Red cell distribution width.

**Table 3:** Comparison of median LAR values according to demographic characteristics and the presence of postoperative complications.

		LAR Median (25th–75th Percentile)	*p*
Demographic characteristics			
Gender	Women Men	5.8 (4.4–7.7) 5.2 (4.3–7.1)	0.16
Comorbidity	(+) (−)	5.4 (4.3–7.2) 5 (4.01–6.2)	0.22
EuroSCORE risk	Low Moderate High	5.3 (4.3–7.1) 6.7 (4.2–8.3) 7.1 (4.3–12.6)	0.46
Postoperative complications			
Postoperative AF	(+) (−)	6.2 (4.5–8.6) 5.3 (4.3–7.1)	0.027
Postoperative VT	(+) (−)	8.9 (7.2–13.3) 5.3 (4.3–7.1)	0.019
Postoperative CVD	(+) (−)	11.4 (6.7–13.6) 5.3 (4.3–7.1)	0.032
Postoperative ARF	(+) (−)	7.8 (5.6–9.7) 5.2 (4.3–7.02)	0.000
Postoperative pneumonia	(+) (−)	6.3 (4.9–8.1) 5.2 (4.3–7.1)	0.007
Mortality			
In-hospital exitus	(+) (−)	8.6 (7.1–13.2) 5.2 (4.3–6.9)	0.000

Abbreviations: *AF:* Atrial fibrillation; *VT:* Ventricular tachycardia; *CVD:* Cerebrovascular disease; *ARF:* Acute renal failure.

**Table 4:** Correlation analysis between LAR and preoperative and postoperative clinical outcomes.

Correlations Spearman’s Rho		Age	Preoperative EF %	LOS-ICU	LOS-Hospital	CPB Time	Cross Clamp Time	Extubation Time
LAR	Correlation Coefficient	0.161 **	−0.227 **	0.17 **	0.208 **	0.065	0.12	0.091
	Sig. (2-tailed)	0.002	0.000	0.001	0.000	0.29	0.058	0.084
	*n*	377	377	377	377	269	269	365

Abbreviations: EF: Ejection fraction; LOS: Length of stay; ICU: Intensive care unit; CPB: Cardiopulmonary bypass. ** Correlation is significant at the 0.01 level (two-tailed)

## Data Availability

Datasets are available on request from the authors.
